# miR-29a-3p inhibits endometrial cancer cell proliferation, migration and invasion by targeting VEGFA/CD C42/PAK1

**DOI:** 10.1186/s12885-021-08506-z

**Published:** 2021-07-21

**Authors:** Aizhi Geng, Lin Luo, Fengyun Ren, Ling Zhang, Haiying Zhou, Xue Gao

**Affiliations:** 1 Department of Gynecology, The Second People’s Hospital of Liaocheng, Liaocheng, 252601 Shandong China; 2 Department of obstetrics and gynecology, People’s Hospital of Rizhao Lanshan, Rizhao, 276807 Shandong China; 3 Department of obstetrics and gynecology, People’s Hospital of Huantai County, Zibo, 256400 Shandong China; 4 Medical Record Room, Gao Qing People’s Hospital, Zibo, 256300 Shandong China; 5 Department of Nursing, Gao Qing People’s Hospital, Zibo, 256300 Shandong China; 6 Department of Obstetrics and Gynecology, Zibo Hospital of Traditional Chinese Medicine, No. 75 Xinajian Middle Road, Zhoucun District, Zibo City, 255300 Shandong China

**Keywords:** Endometrial cancer, miR-29a-3p, VEGFA, CDC42/PAK1 signaling, Progression

## Abstract

**Background:**

This study aimed to investigate the mechanism of miR-29a-3p in regulating endometrial cancer (EC) progression.

**Methods:**

A total of 72 EC patients were enrolled. EC cells were transfected. Cells proliferation, cloning ability, migration and invasion were researched by MTT assay, colony formation experiment, cell scratch test and Transwell experiment respectively. Dual-luciferase reporter assay was performed. Xenograft experiment was conducted using nude mice. miR-29a-3p, VEGFA, CDC42, PAK1 and p-PAK1 expression in cells/tissues was investigated by qRT-PCR and Western blot.

**Results:**

miR-29a-3p expression was aberrantly reduced in EC patients, which was associated with poor outcome. miR-29a-3p inhibited EC cells proliferation, cloning formation, migration and invasion (*P* <  0.05 or *P* <  0.01 or *P* <  0.001). miR-29a-3p inhibited CDC42/PAK1 signaling pathway activity in EC cells (*P* <  0.01). VEGFA expression was directly inhibited by miR-29a-3p. miR-29a-3p suppressed EC cells malignant phenotype in vitro and growth in vivo by targeting VEGFA/CDC42/PAK1 signaling pathway (*P* < 0.05 or *P* < 0.01).

**Conclusion:**

miR-29a-3p inhibits EC cells proliferation, migration and invasion by targeting VEGFA/CDC42/PAK1 signaling pathway.

**Supplementary Information:**

The online version contains supplementary material available at 10.1186/s12885-021-08506-z.

## Background

Endometrial cancer (EC) is a common gynecological malignancy which accounts for about 4.8% of cancers in women [1]. Invasion or metastasis is one of the main causes of malignant development of EC. EC patients diagnosed at early stage have a 5-year survival rate of approximately 82% [2]. For EC patients diagnosed at advanced stage, the 5-year survival rate is less than 30% [3]. EC patients with advanced stage often have worse response to the traditional treatment strategies, such as surgical resection adjuvant chemotherapy and radiotherapy [2]. However, chemotherapy and radiotherapy often cause harmful effects on healthy tissues and organs [4, 5]. Therefore, understanding the internal molecular mechanism of EC is of great significance for finding targeted therapy and improving the therapeutic effect and prognosis of EC patients.

With the development of molecular biology, biomedical informatics and novel diagnostic technologies have become an essential component in medical research [6, 7]. In recent years, one of the hot spots in the field of genetics was to explore microRNAs (miRNAs) as potential biomarkers and target for diseases diagnosis and treatment [8, 9]. miRNAs are a class of small non-coding RNAs that regulate gene expression at the post-transcriptional level [10]. It participates in a series of biological processes in human disease development, particularly in tumorigenesis and progression. miR-29a-3p has been found to be abnormally reduced in multiple human malignant tumors. It acts as an important tumor suppressor in laryngocarcinoma, papillary thyroid carcinoma, hepatocellular carcinoma and gastric cancer [11–14]. In gynecological cancers, miR-29a-3p was found to be involved in breast cancer progression. Previous studies have revealed circRNA ACAP2 (circACAP2) promoted the metastasis and proliferation of breast cancer by sponging miR-29a-3p [15]. In ovarian cancer, the up-regulation of miR-29a-3p could suppress ovarian cancer cells invasion and proliferation [16]. Moreover, the function of miR-29a-3p in EC progression has not yet been clarified. Based on the above previous data, it was speculated that miR-29a-3p might act as a tumor suppressor in EC. Thus, this study was designed to explore the function of miR-29a-3p in the development of EC. This is the first time that the function of miR-29a-3p has been studied in EC.

One of the main ways that miRNAs regulate tumor development is to regulate coding genes expression at the post-transcriptional level [17]. Previous research have reported that abnormal expression of vascular endothelial growth factor A (VEGFA) is involved in human tumors progression [18]. This study used TargetScan online analysis and observed that miR-29a-3p and VEGFA have common binding site in the 3′-UTR region. Therefore, it was speculated that VEGFA might be a target of miR-29a-3p and miR-29a-3p might regulate EC progression by targeting VEGFA. CDC42/PAK1 signaling pathway has been found to be one of the important factors in tumor development [19]. At present, the links between miR-29a-3p and CDC42/PAK1 signaling pathway have not been studied. This study speculated that miR-29a-3p might affect EC development by regulating the VEGFA/CDC42/PAK1 signaling pathway. This study might provide a novel target and precise molecular mechanism for EC treatment. The objectives of this research were to detect miR-29a-3p expression and function in EC progression. miR-29a-3p effects on EC cells proliferation, migration, invasion and CDC42/PAK1 signaling pathway was researched. Dual-luciferase reporter assay was performed to detect the relationship between miR-29a-3p and VEGFA. The internal mechanism of miR-29a-3p and VEGFA in regulating EC development was verified by in vitro rescue experiment and in vivo xenograft tumor experiment.

## Methods

### Clinical tissues collection

Tumor tissues of 72 EC patients were obtained. These patients were diagnosed with EC for the first time in our hospital from March 2014 to January 2015. All patients were received surgical resection. Adjacent normal tissues of 30 patients with EC were obtained and used as control. All patients have no previous history of cancer-related treatment. All the specimens were immediately stored in liquid nitrogen after obtained. The clinicopathological characteristics of the 72 patients were listed in Table 1, including age, differentiation, FIGO stage, cancer type, muscular infiltration and lymphatic metastasis. The relationship between miR-29a-3p expression and clinicopathological characteristics was analyzed.

**Table 1:** The relationship between miR-29a-3p expression in EC tissues and clinicopathological characteristics of patients

Characteristics	Number(*n* = 72)	Relative miR-29a-3p expression	*P* value
Age (years)			0.2910
< 50	36	0.533 ± 0.107	
≥ 50	36	0.504 ± 0.132	
Differentiation			0.001**
High and mediumdifferentiation	31	0.587 ± 0.106	
Low and undifferentiation	41	0.467 ± 0.102	
FIGO stage			< 0.0001 ***
I-II	43	0.566 ± 0.098	
III-IV	29	0.448 ± 0.116	
Cancer type			0.9093
Type I	24	0.516 ± 0.131	
Type II	48	0.520 ± 0.116	
Muscular infiltration			0.0002 ***
< 1/2	34	0.572 ± 0.103	
≥ 1/2	38	0.471 ± 0.115	
Lymphatic metastasis			< 0.0001 ***
No	28	0.548 ± 0.124	
Yes	44	0.423 ± 0.115	

**P < 0.01 and ***P < 0.001. Note: Type I indicated patientswithestrogen receptor-dependent EC. Type II indicated patients with non-estrogen receptor-dependent EC

All participants in this study have voluntarily signed written informed consent. This study has been approved by the Second People’s Hospital of Liaocheng Ethics Committee in line with the Declaration of Helsinki.

### Cell culture

Human EC cell lines (HEC-1A and Ishikawa) and endometrial epithelial cell line (EEC) were obtained from the American Type Culture Collection (ATCC, Rockville, MD, USA). Cells were maintained in RPMI-1640 medium suspended with 10% fetal bovine serum (FBS), 100 U/mL penicillin and 100 μg/mL streptomycin in an incubator at 37 °C, 5% CO_2_.

### Cell transfection and grouping

HEC-1A and Ishikawa cells in the logarithmic growth phase were harvested. These cells were prepared into cell suspension with serum-free RPMI-1640 medium (1 × 10^6^ cells/mL). A total of 1 mL cell suspension was seeded in 6-well plates. miR-29a-3p mimic and corresponding negative control (GenePharma, Shanghai, China) were respectively transfected into HEC-1A and Ishikawa cells (set as miR-29a-3p mimic group and miR-NC group, respectively). The full-length sequence of VEGFA was synthesized (GenePharma, Shanghai, China) and cloned into pcDNA3.1 plasmid (Invitrogen, Carlsbad, CA, USA) according to the instructions. HEC-1A and Ishikawa cells were subjected to cotransfection with miR-29a-3p mimic and pcDNA3.1-VEGFA plasmid (named miR-29a-3p mimic + pcDNA-VEGFA group). All transfection operations were performed strictly in line with the instruction of Lipofectamine 3000 (Thermo Fisher Scientific, Waltham, MA, USA). Cells were kept at 37 °C, 5% CO_2_ for 8 h. Subsequently, the residual liquid in each well was discarded. RPMI-1640 medium containing 10% FBS was used to culture cells for 48 h. HEC-1A and Ishikawa cells without transfection were cultured in RPMI-1640 medium containing 10% FBS (served as BLANK group).

### MTT assay

HEC-1A and Ishikawa cells were dispersed in RPMI-1640 medium containing 10% FBS (5 × 10^4^ cells/mL). The cell suspension was inoculated into 96-well plates (100 μL per well). All 96-well plates were incubated at 37 °C, 5% CO_2_ for 0, 24, 48, 72 and 96 h, respectively. Thereafter, 3-(4,5-dimethylthiazol-2-yl)-2,5-diphenyltetrazolium bromide (MTT) solution (5 mg/mL, 20 μL) was added into each well to incubate cells for 4 h at 37 °C. After the residual liquid in each well being discarded, 150 μL dimethyl sulfoxide (DMSO) was added into each well. The 96-well plates were gently shaken for 10 min. The optical density (OD) value of each well was determined using a microplate reader (Bio-Tek Instruments, Winooski, VT, USA) at 570 nm. For cells in each group, 5 multiple wells were set.

### Colony formation experiment

HEC-1A and Ishikawa cells were collected after 48 h of transfection, followed by being washed with phosphate-buffered saline (PBS). Totally 300 cells were seeded into 6-well plates. Cells were incubated with 2 mL of RPMI-1640 medium containing 10% FBS at 37 °C, 5% CO_2_. The medium in each well was changed every 3 days. Cells were cultured for 14 consecutive days. Then the residual liquid in each well was discarded. Cells attached to the bottom of the well plate were fixed with 4% paraformaldehyde for 15 min at room temperature. Afterward, 0.1% crystal violet was added to stain cells for 10 min at room temperature. Under an inverted microscope (Olympus CK40, Tokyo, Japan), the number of clones was automatically counted using Image J software (National Institutes of Health, Bethesda, MD, USA).

### Cell scratch test

HEC-1A and Ishikawa cells were seeded in 6-well plates. In each well, 1 × 10^5^ cells dispersed in 1 mL RPMI-1640 medium (10% FBS) was contained. Cells were kept at 37 °C, 5% CO_2_ for 24 h. Thereafter, a diameter was drawn at the bottom of each well using a 200 μL sterile pipette tip. The scratch width was measured and recorded as the scratch width of 0 h. The residual liquid in each well was replaced by fresh RPMI-1640 medium (10% FBS). Cells were then cultured at 37 °C, 5% CO_2_ for 24 h. The scratch width was measured again, and was recorded as the scratch width of 24 h. The migration rate was calculated using the formula of (the scratch width of 0 h- the scratch width of 24 h) × 100%/the scratch width of 0 h.

### Transwell experiment

The upper membrane of the Transwell chambers (8 μm pore size) was covered with a layer of Matrigel (1 mg/mL, 100 μL). The Transwell chambers were inserted into 24-well plates containing 500 μL RPMI-1640 medium suspended with 10% FBS. A total of 2 × 10^5^ HEC-1A and Ishikawa cells were dispersed in 200 μL serum-free RPMI-1640 medium. Cells were seeded into the upper chamber. After being maintained at 37 °C, 5% CO_2_ for 24 h, the Transwell chambers were removed. Cells on the upper membrane were gently removed with a cotton swab. Cells invading through the membrane were fixed with 4% paraformaldehyde for 15 min at room temperature. Staining of cells was performed using 0.1% crystal violet for 10 min. The number of invading cells was counted with 6 random non-overlapping fields of view under a microscope (Olympus, Tokyo, Japan) (200 × magnification).

### qRT-PCR

Tissues were ground into powder in liquid nitrogen. Cells were collected after 48 h of transfection. According to the instructions, total RNA in tissues powder and cells were extracted using TRIzol reagent (Solarbio, Beijing, China). The cDNA template was obtained by PrimeScript RT reagent Kit (Takara, Otsu, Shiga, Japan) using 5 μg of the total RNA extracts. PCR was carried out with SYBR Green reagent (TaKaRa, Otsu, Shiga, Japan) on the ABI 7500 fast real-time PCR System (Applied Biosystems, Foster City, CA, USA). The parameters of PCR was 5 min at 95 °C, followed by 40 cycles of 15 s at 95 °C, 30 s at 55 °C, and 30 s at 72 °C. Primers were as follows: miR-29a-3p, forward, 5′-CTGGTGTCGTGGAATTCAGTTGA-3′, reverse, 5′-CCTGGCTCCTCACTTGGC-3′. U6, forward, 5′-CTCGCTTCGGCAGCACA-3′, reverse, 5′-AACGCTTCACGAATTTGCGT-3′. VEGFA, forward: 5′-TGGCTCACTGGCTTGCTCTA-3′, reverse: 5′-ATCCAACTGCACCGTCACAG-3′. GAPDH, forward: 5′-GGTGGTCTCCTCTGACTTCAA-3′, reverse: 5′-GTTGCTGTAGCCAAATTCGTTGT-3′. U6 was used as the control for miR-29a-3p and GAPDH was set as the control for VEGFA. The 2^-ΔΔCt^ method was used for the analysis of relative miR-29a-3p and VEGFA mRNA expression.

### Dual-luciferase reporter assay

VEGFA possessed binding site for miR-29a-3p in the 3 as the contraccording to TargetScan (http://www.targetscan.org/vert_71/). The 3(http segments of VEGFA, including mutant type (Mut) and wild type (Wt), were designed and synthesized (GenePharma, Shanghai, China). These segments were cloned into the pmirGLO luciferase reporter (Promega, Madison, WI, USA) according to the instructions. HEC-1A and Ishikawa cells of miR-NC group and miR-29a-3p mimic group were seeded in 6-well plates with serum-free RPMI-1640 medium. Then cells were cotransfected by pmirGLO-VEGFA-Mut luciferase reporter or pmirGLO-VEGFA-Wt luciferase reporter. Cells were kept at 37 °C, 5% CO_2_ for 8 h. RPMI-1640 medium with 10% FBS was then used to incubate cells for 48 h. Dual-Glo luciferase assay kit (Promega, Madison, WI, USA) was applied for the detection of luciferase activity.

### Xenograft experiment

Animal experiments in this research have been approved by the Second People’s Hospital of Liaocheng Ethics Committee.

Female nude mice (*n* = 9, 4–5 weeks old) were commercially obtained from Shanghai Experimental Animal Center, Chinese Academy of Sciences (Shanghai, China). Mice were housed in a room at 22–25 °C and 50–65% humidity with free access to food and water. HEC-1A cells of miR-NC group, miR-29a-3p mimic group and miR-29a-3p mimic + pcDNA-VEGFA group were collected and dispersed in PBS (1 × 10^6^ cells/mL). Totally 100 μL of each cell suspension was injected subcutaneously into the right back of nude mice. After injection, mice were kept for 4 weeks with free access to food and water. Every week, the long (a) and short (b) diameters of the tumor tissues were measured. The tumor volume was calculated with the formula of 0.5 * a * b^2^. On the 4th week, all mice were euthanized. The xenograft tumor tissues were stripped and weight. Xenograft tumor tissues were immediately stored in liquid nitrogen for the following researches.

The procedure for euthanasia of nude mice was as follows: mice were deeply anesthetized by intraperitoneal injection of pentobarbital at a dose of 60 mg/kg. Thereafter, mice were quickly sacrificed by dislocating the neck. The standard for deep anesthesia was unresponsiveness of nude mice to limb and head stimulation.

### Western blot

Tissues stored in liquid nitrogen were ground into powder. HEC-1A and Ishikawa cells were collected after 48 h of transfection. RIPA lysis buffer (Solarbio, Beijing, China) was added into the tissues powder and cells to extract total protein. The total protein concentration was investigated by BCA kit (Beyotime, Shanghai, China). For protein separation, 10% sodium dodecyl sulphate-polyacrylamide gel electrophoresis (SDS-PAGE) was performed with 10 μL of total protein samples. The separated protein was transferred onto polyvinylidene fluoride (PVDF) membrane. Then 5% skimmed milk was added to block protein for 1 h. Primary antibodies was used to probe protein for 12 h at 4 °C. The primary antibodies used in this study were as follows: rabbit anti-PAK1, anti-p-PAK1 and anti-VEGFA (1:1000, Abcam, Cambridgeshire, UK), rabbit anti-CDC42 and anti-GAPDH (1:1000, Cell Signaling, Beverly, MA, USA). The PVDF membrane was then treated by goat anti-rabbit secondary antibody (1:5000, Solarbio, Beijing, China) for 1 h at room temperature. The protein blots were visualized using enhanced chemiluminescence (ECL) system (Pierce Biotechnology, Rockford, IL, USA) according to the instructions. Image J software (NIH, Bethesda, MD, USA) was used for the blots intensity detection. GAPDH was the internal control.

### Statistical analysis

All experiments were independently conducted at least 3 times. Data were exhibited as mean ± standard deviation and analyzed by SPSS 19.0 software (SPSS, Inc., Chicago, IL, USA). Comparison between two groups was analyzed by Student’s t-test. Comparison at least in three groups was assessed by one-way analysis of variance (ANOVA). *P* < 0.05 was indicated statistically significant difference.

## Results

### miR-29a-3p expression was reduced in EC tissues of patients

**Fig. 1:**
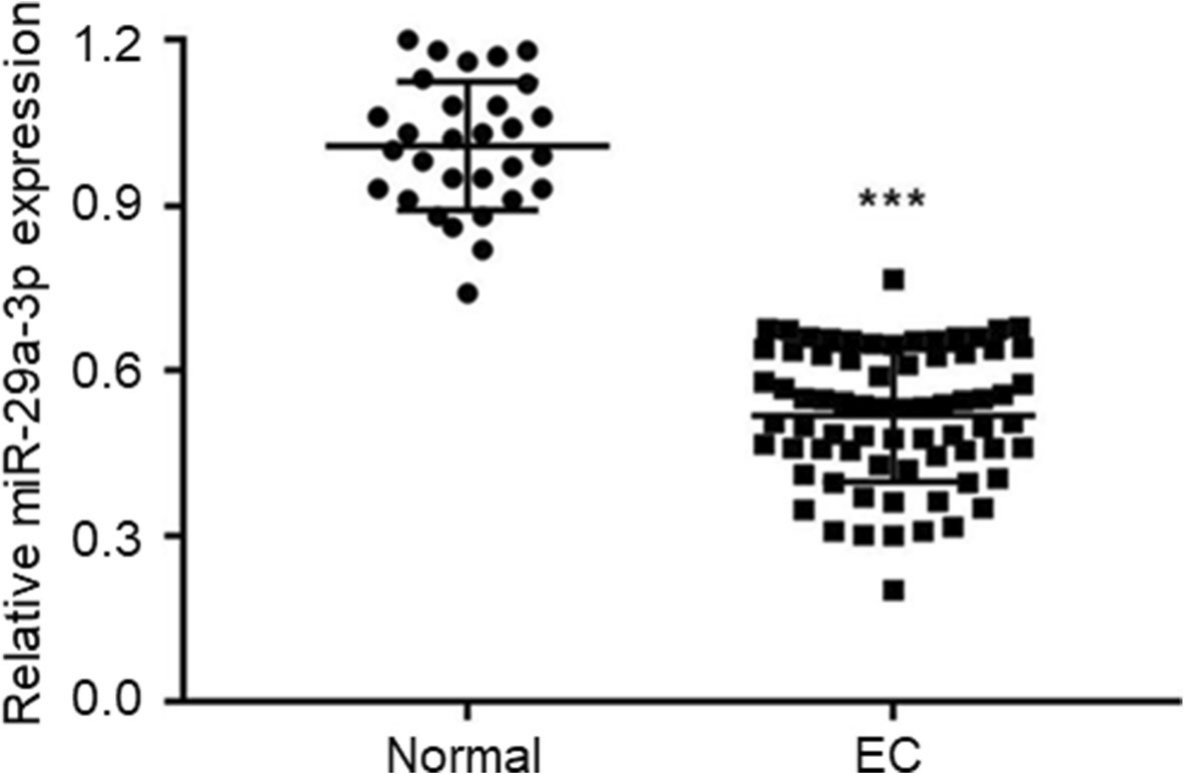
miR-29a-3p expression was reduced in EC tissues of patients. ***P < 0.001

qRT-PCR showed aberrantly reduced miR-29a-3p expression in EC tissues than that in adjacent normal tissues (*P* < 0.001) (Fig. 1). Thus, miR-29a-3p expression was reduced in EC patients. The relationship between miR-29a-3p expression in EC tissues and clinicopathological characteristics of patients was analyzed. As listed in Table 1, low miR-29a-3p expression was obviously associated with poor prognosis of EC patients, including low differentiation and undifferentiation, advanced FIGO stage, severe muscular infiltration and positive lymphatic metastasis (*P* < 0.01 or *P* < 0.001). These data indicated that low miR-29a-3p expression was associated with poor outcome of patients.

### miR-29a-3p inhibited EC cells proliferation and colony formation

**Fig. 2:**
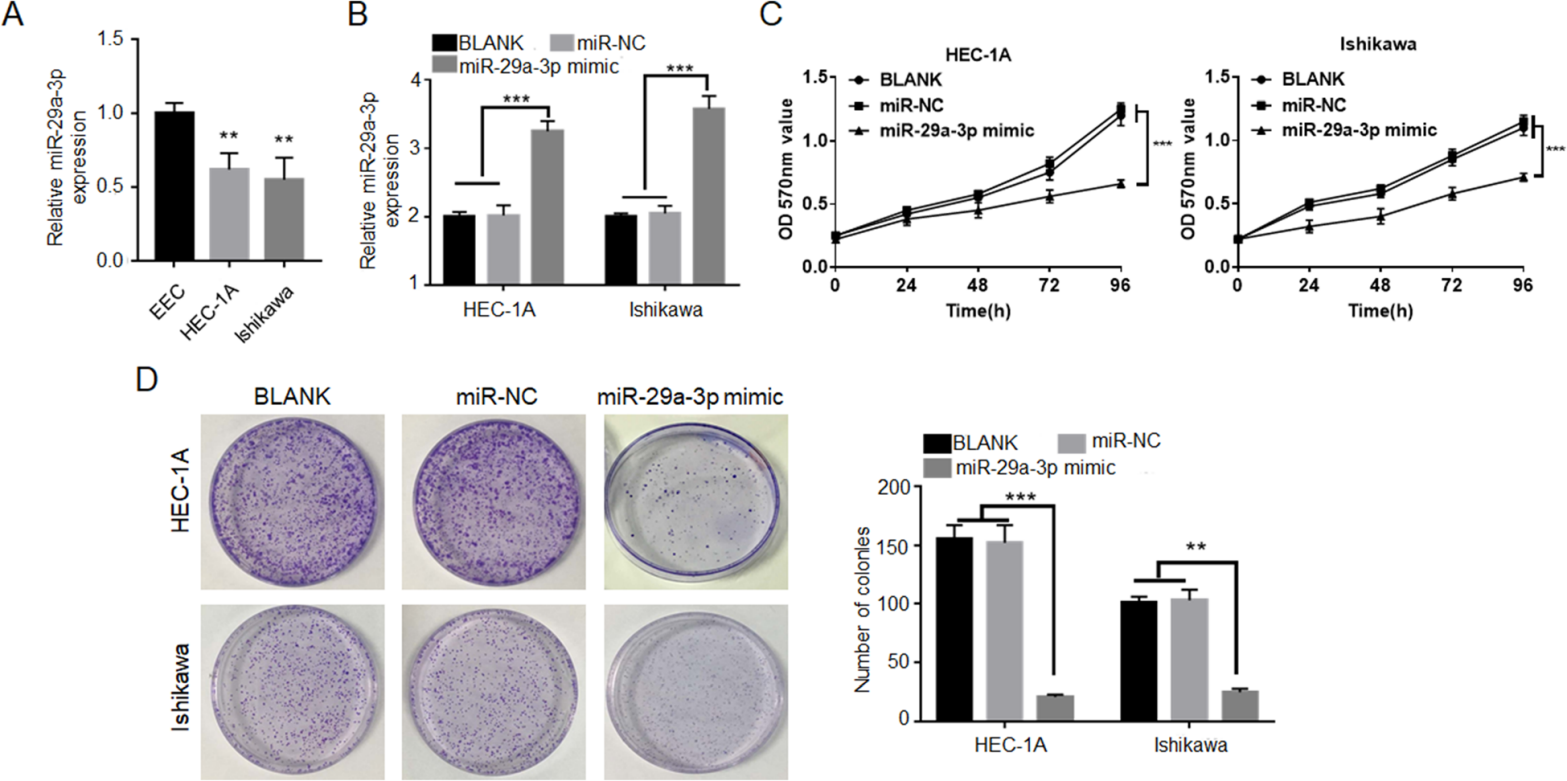
miR-29a-3p inhibited EC cells proliferation and colony formation. **A** EC cells (HEC-1A and Ishikawa) expressed markedly lower miR-29a-3p expression than EEC. ** *P* < 0.01 relative to miR-29a-3p expression in EEC. **B** miR-29a-3p expression was successfully up-regulated in HEC-1A and Ishikawa cells by transfection of miR-29a-3p mimic. **C** miR-29a-3p up-regulation inhibited HEC-1A and Ishikawa cells proliferation according to MTT assay. **D** Colony formation experiment indicated thatmiR-29a-3p up-regulation inhibited HEC-1A and Ishikawa cells colony formation ability. ** *P* < 0.01 and *** *P* < 0.001 relative to BLANK groupandmiR-NC group

qRT-PCR exhibited that, EC cells (HEC-1A and Ishikawa) had markedly lower miR-29a-3p expression than EEC cells (*P* < 0.01) (Fig. 2A). HEC-1A and Ishikawa cells were transfected by miR-29a-3p mimic and negative control. qRT-PCR showed that HEC-1A and Ishikawa cells of miR-29a-3p mimic group exhibited dramatically higher miR-29a-3p expression than that of BLANK group and miR-NC group (*P* < 0.001) (Fig. 2B). This revealed that the expression of miR-29a-3p in HEC-1A and Ishikawa cells was successfully up-regulated by transfection. MTT assay presented remarkably lower OD570 value of HEC-1A and Ishikawa cells in miR-29a-3p mimic group when compared with BLANK group and miR-NC group (*P* < 0.001) (Fig. 2C). According to colony formation experiment, significantly less number of colonies was found in HEC-1A and Ishikawa cells of miR-29a-3p mimic group relative to BLANK group and miR-NC group (*P* < 0.01 or *P* < 0.001) (Fig. 2D). Therefore, miR-29a-3p inhibited EC cells proliferation and colony formation.

### miR-29a-3p inhibited EC cells migration and invasion

**Fig. 3:**
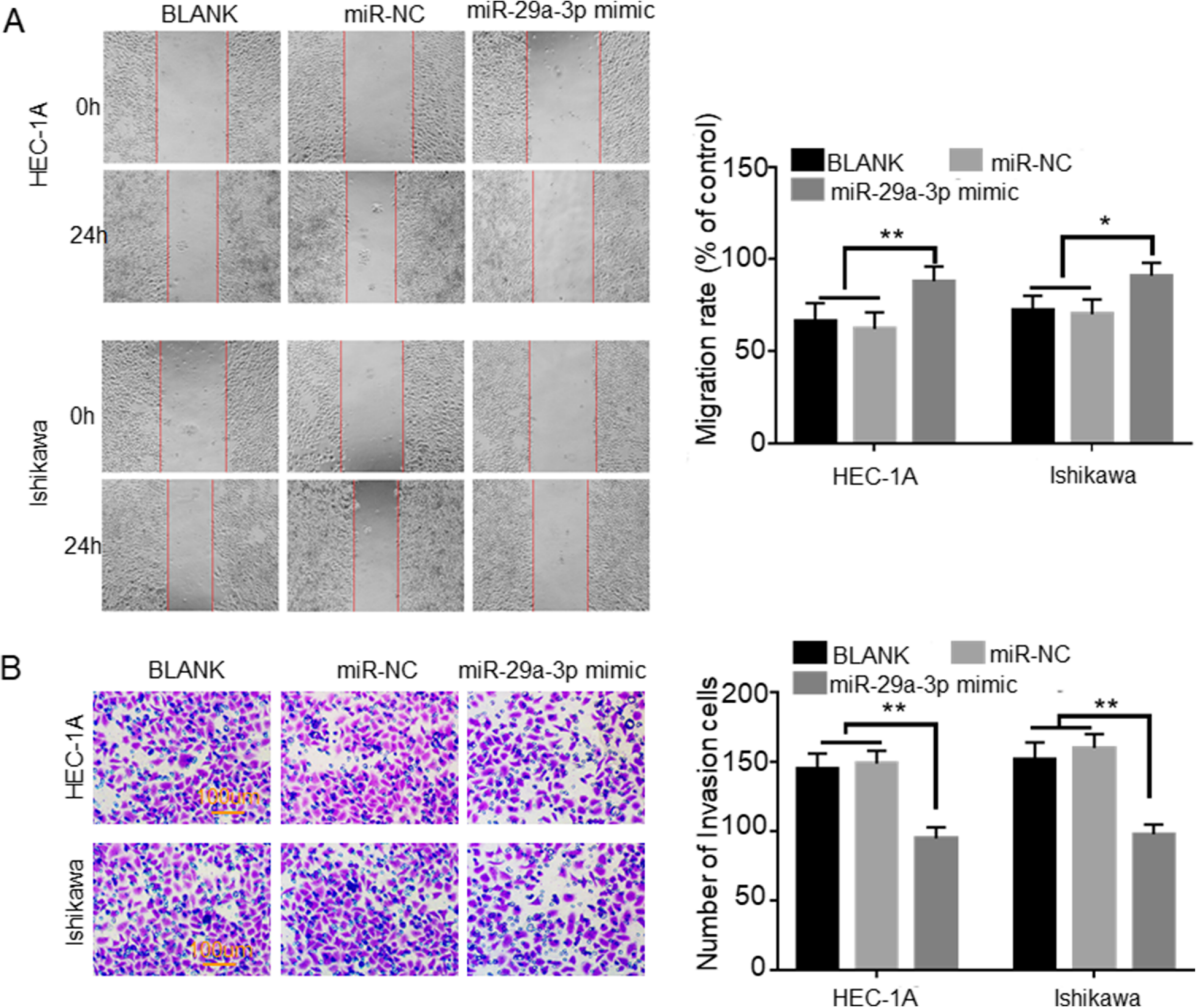
miR-29a-3p inhibited EC cells migration and invasion. **A** miR-29a-3p up-regulation inhibited EC cells migration. **B** miR-29a-3p up-regulation inhibited EC cells invasion. * *P* < 0.05 and ** *P* < 0.01 relative to BLANK groupandmiR-NC group

Cell scratch test exhibited that, comparison with BLANK group and miR-NC group, HEC-1A and Ishikawa cells of miR-29a-3p mimic group presented much higher migration rate (*P* < 0.05 or *P* < 0.01) (Fig. 3A). Transwell experiment was performed to detect invasion ability. More number of invasion HEC-1A and Ishikawa cells was observed in miR-29a-3p mimic group when relative to BLANK group and miR-NC group (*P* < 0.01) (Fig. 3B). These data revealed that miR-29a-3p inhibited EC cells migration and invasion.

### miR-29a-3p inhibited the activity of CDC42/PAK1 signaling pathway in EC cells

**Fig. 4:**
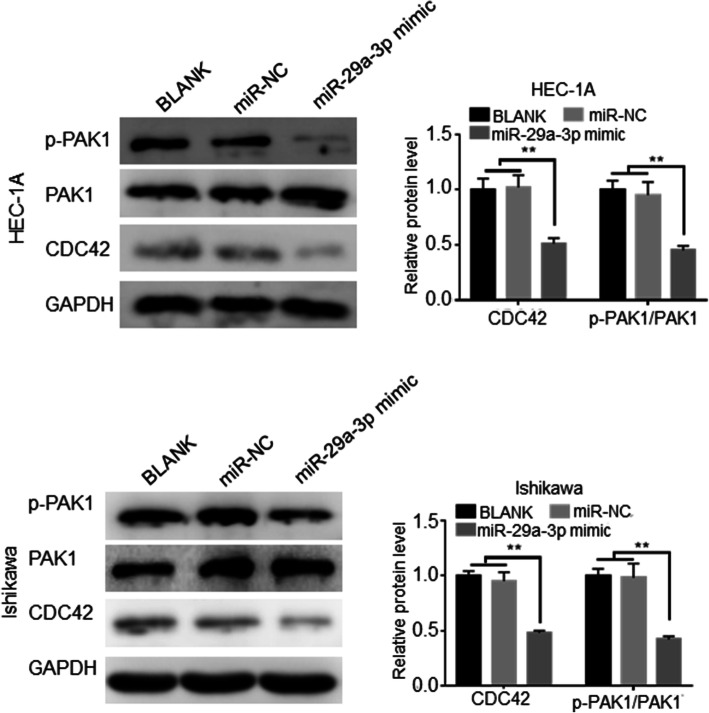
miR-29a-3p inhibited the activity of CDC42/PAK1 signaling pathway in EC cells. ** *P* < 0.01 relative to BLANK groupandmiR-NC group

This study explored the effect of miR-29a-3p on the activity of CDC42/PAK1 signaling pathway. The results were shown in Fig. 4. Compared to BLANK group and miR-NC group, dramatically decreased CDC42 and p-PAK1/PAK1 protein expression was observed in HEC-1A and Ishikawa cells of miR-29a-3p mimic group (*P* < 0.01). Thus, miR-29a-3p inhibited the expression of CDC42 and suppressed the phosphorylation of PAK1. This illustrated that miR-29a-3p inhibited the activity of CDC42/PAK1 signaling pathway in EC cells.

### VEGFA was a target gene of miR-29a-3p, which expression was directly inhibited by miR-29a-3p

**Fig. 5:**
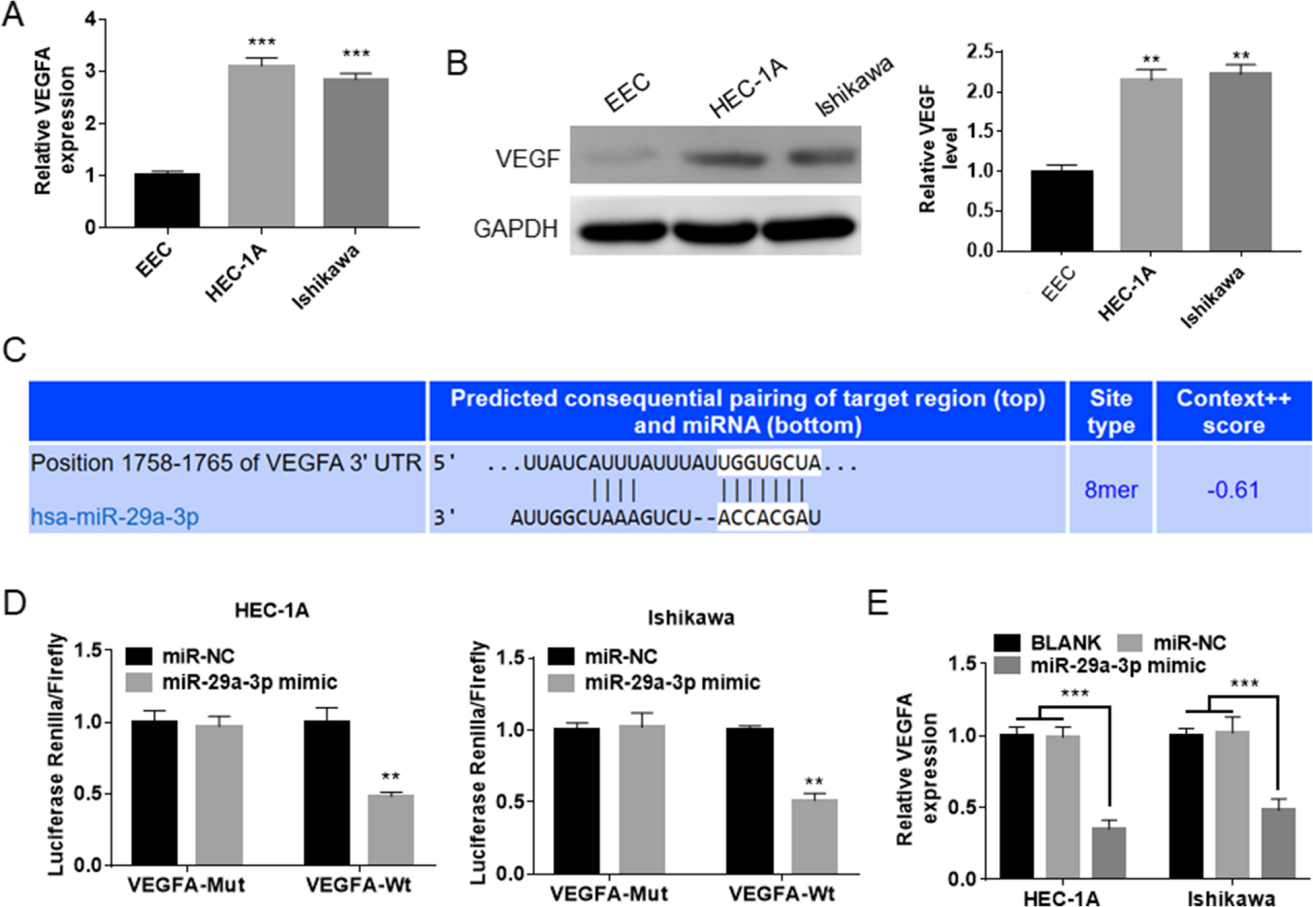
VEGFA was a target gene of miR-29a-3p, which expression was directly inhibited by miR-29a-3p. **A** VEGFA mRNA expression was increased in HEC-1A and Ishikawa cells when compared withthat in EEC. *** *P* < 0.001 when relative to VEGFA mRNA expression in EEC. **B** VEGFA protein expression was elevated in HEC-1A and Ishikawa cells when relative to that in EEC. ** *P* < 0.01 when relative to VEGFA protein expression in EEC. **C** Prediction from TargetScan showed thatVEGFA possessed binding site for miR-29a-3p in the 3C-1A and Ishikawa cellsluciferase reporter gene assay verified that VEGFA was directly inhibited by miR-29a-3p. ** *P* < 0.01 when relative to miR-NC group. **E** miR-29a-3p up-regulation reduced VEGFA mRNA expression in HEC-1A and Ishikawa cells. *** *P* < 0.001 relative to BLANK groupandmiR-NC group

Prominently higher VEGFA mRNA and protein expression was found in HEC-1A and Ishikawa cells when compared with that in EEC (*P* < 0.01 or *P* < 0.001) (Fig. 5AB). This data suggested that VEGFA was up-regulated in EC cells. TargetScan showed that VEGFA possessed binding site for miR-29a-3p in the 3′-UTR region (Fig. 5C). Therefore, it was speculated that VEGFA might be a target of miR-29a-3p. Based on this speculation, dual luciferase reporter gene assay was conducted to verify the relationship between miR-29a-3p and VEGFA. As a result, relative to miR-NC group, there were not significant changes in the pmirGLO-VEGFA-Mut luciferase reporter activity in HEC-1A and Ishikawa cells of miR-29a-3p mimic group. However, HEC-1A and Ishikawa cells of miR-29a-3p mimic group presented distinctly lower luciferase activity of pmirGLO-VEGFA-Wt luciferase reporter (*P* < 0.01) (Fig. 5D). These data revealed that VEGFA was target gene of miR-29a-3p. qRT-PCR showed that, compared with the VEGFA mRNA expression in HEC-1A and Ishikawa cells of BLANK group and miR-NC group, it was remarkably declined in miR-29a-3p mimic group (*P* < 0.001) (Fig. 5E). These data further indicated that VEGFA expression was directly inhibited by miR-29a-3p.

### miR-29a-3p suppressed EC cells proliferation, colony formation, migration and invasion by inhibiting CDC42/PAK1 signaling pathway activity via targeting VEGFA

**Fig. 6:**
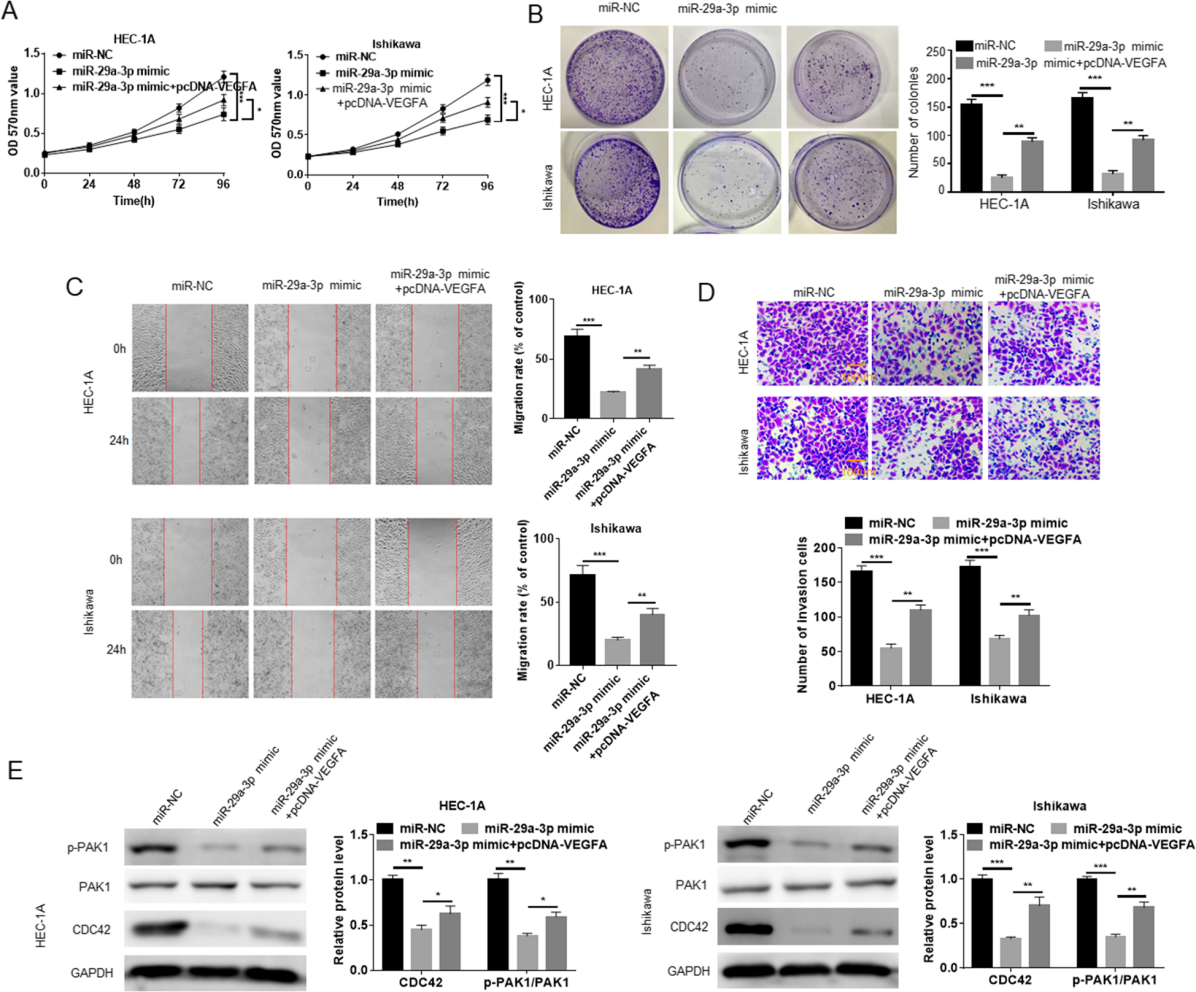
miR-29a-3p suppressed EC cells proliferation, colony formation, migration and invasion by inhibiting CDC42/PAK1 signaling pathway activity via targeting VEGFA. **A** VEGFA up-regulation reversed the inhibitory effect of miR-29a-3p on EC cells proliferation. **B** VEGFA up-regulation reversed the inhibitory effect of miR-29a-3p on EC cells colony ability. **C** VEGFA up-regulation reversed the inhibitory effect of miR-29a-3p on EC cells migration. **D** VEGFA up-regulation reversed the inhibitory effect of miR-29a-3p on EC cells invasion. **E** VEGFA up-regulation reversed the inhibitory effect of miR-29a-3p on CDC42/PAK1 signaling pathway activity. * *P* < 0.05, ** *P* < 0.01 and *** *P* < 0.001

HEC-1A and Ishikawa cells were experienced co-transfection to detect the malignant phenotype in vitro. MTT assay showed that, HEC-1A and Ishikawa cells of miR-29a-3p mimic group had much lower OD570 value than that of miR-NC group (*P* < 0.001). However, relative to miR-29a-3p mimic group, the OD570 value of HEC-1A and Ishikawa cells in miR-29a-3p mimic + pcDNA-VEGFA group was significantly elevated (*P* < 0.05) (Fig. 6A). Based on colony formation experiment, much lower number of colonies was found in HEC-1A and Ishikawa cells of miR-29a-3p mimic group when compared with miR-NC group (*P* < 0.001). Conversely, HEC-1A and Ishikawa cells of miR-29a-3p mimic + pcDNA-VEGFA group exhibited obviously higher number of colonies than that of miR-29a-3p mimic group (*P* < 0.05) (Fig. 6B). Cell scratch test and Transwell experiment presented that, relative to miR-NC group, the migration rate and number of invasion cells was both markedly reduced in HEC-1A and Ishikawa cells of miR-29a-3p mimic group (*P* < 0.001). Oppositely, in comparison with miR-29a-3p mimic group, prominently increased migration rate and number of invasion cells was observed in HEC-1A and Ishikawa cells of miR-29a-3p mimic + pcDNA-VEGFA group (*P* < 0.01) (Fig. 6CD). According to Western blot, significantly lower CDC42 and p-PAK1/PAK1 protein expression was found in HEC-1A and Ishikawa cells of miR-29a-3p mimic group when relative to miR-NC group (*P* < 0.01 or *P* < 0.001). However, in comparison with miR-29a-3p mimic group, the expression of CDC42 and p-PAK1/PAK1 protein was remarkably elevated in HEC-1A and Ishikawa cells of miR-29a-3p mimic + pcDNA-VEGFA group (*P* < 0.05 or *P* < 0.01) (Fig. 6E). These results revealed that miR-29a-3p suppressed EC cells proliferation, colony formation, migration and invasion by inhibiting CDC42/PAK1 signaling pathway activity via targeting VEGFA.

### miR-29a-3p suppressed EC cells growth in vivo by inhibiting CDC42/PAK1 signaling pathway activity via targeting VEGFA

**Fig. 7:**
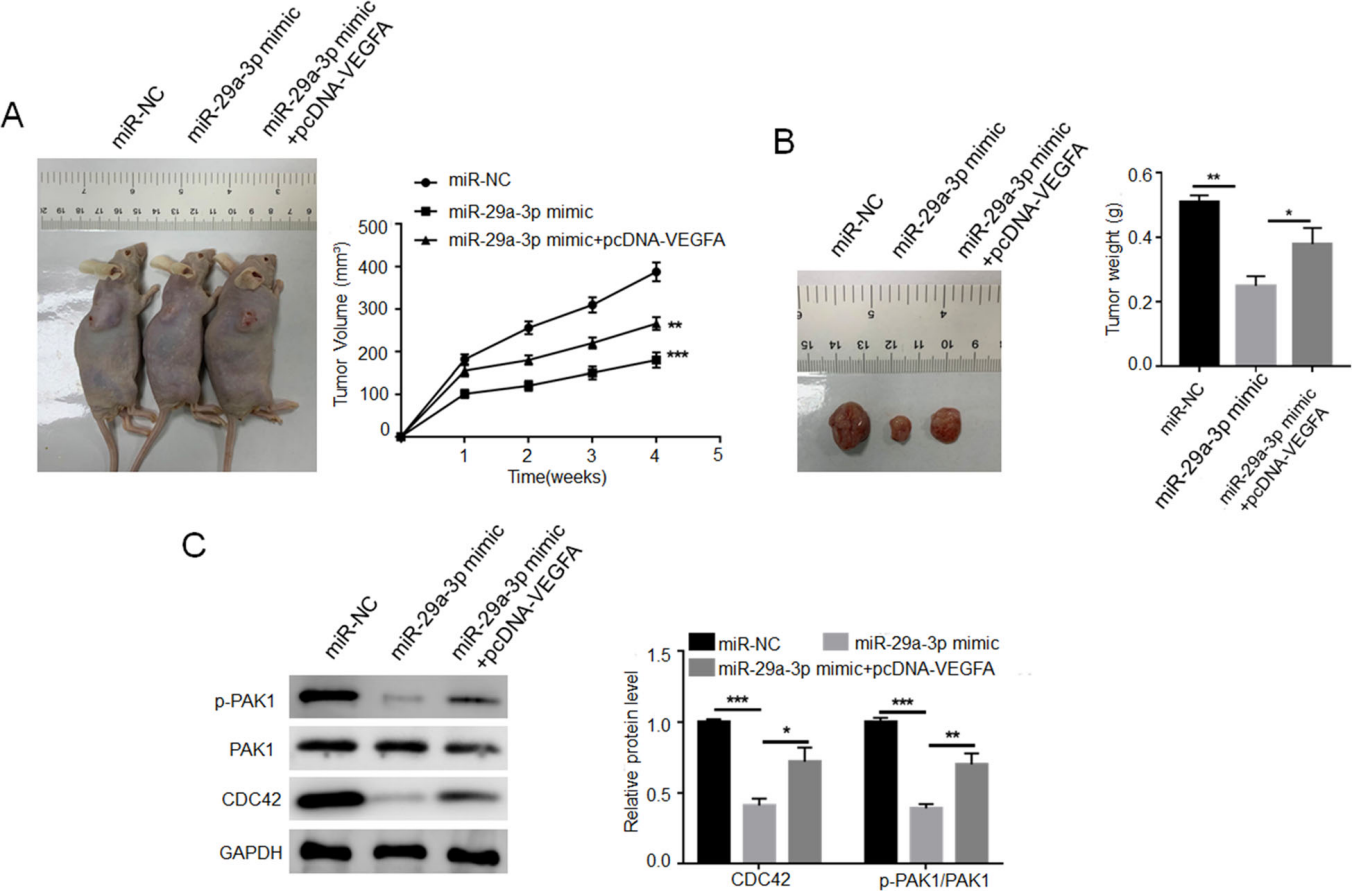
miR-29a-3p suppressed EC cells growth in vivo by inhibiting CDC42/PAK1 signaling pathway activity via targeting VEGFA. **A** VEGFA up-regulation reversed the inhibitory effect of miR-29a-3p on xenograft tumors volume in vivo. ** *P* < 0.01 relative to miR-29a-3p mimic group. *** *P* < 0.001 relative to miR-NC group. **B** VEGFA up-regulation reversed the inhibitory effect of miR-29a-3p on xenograft tumor weight in vivo. * *P* < 0.05 and ** *P* < 0.01. **C** VEGFA up-regulation reversed the inhibitory effect of miR-29a-3p on the expression of CDC42 and p-PAK1/PAK1 protein in xenograft tumors. * *P* < 0.05, ** *P* < 0.01 and *** *P* < 0.001

In vivo study was performed by subcutaneous injecting HEC-1A cells into nude mice. As shown in Fig. 7AB, the xenograft tumors volume and weight was prominently lower in mice of miR-29a-3p mimic group when relative to miR-NC group (*P* < 0.01 or *P* < 0.001). However, in comparison with miR-29a-3p mimic group, markedly higher xenograft tumors volume and weight was observed in mice of miR-29a-3p mimic + pcDNA-VEGFA group (*P* < 0.05 or *P* < 0.01). Western blot exhibited much decreased CDC42 and p-PAK1/PAK1 protein expression in xenograft tumors of miR-29a-3p mimic group than that of miR-NC group (*P* < 0.001). On the opposite, the expression of CDC42 and p-PAK1/PAK1 protein was obviously increased in xenograft tumors of miR-29a-3p mimic + pcDNA-VEGFA group when relative to miR-29a-3p mimic group (*P* < 0.05 or *P* < 0.01) (Fig. 7C). All of these data revealed that miR-29a-3p suppressed EC cells growth in vivo by inhibiting CDC42/PAK1 signaling pathway activity via targeting VEGFA.

## Discussion

miRNAs are endogenous non-coding single-stranded small RNA molecule with about 22 nucleotides in length, which mainly bound to the target mRNA in the 3′-UTR region [20]. In EC, the function of several miRNAs has been elucidated. For example, miR-652, miR-940 and miR-21-5p were identified as cancer-promoting miRNAs for EC [21–23], whereas miR-137, miR-135a and miR-214-3p were considered as tumor suppressor in EC [24–26]. The elucidation of these miRNAs function provided more effective options for EC target therapy. In this study, low miR-29a-3p expression was significantly associated with poor prognosis of EC patients, such as low differentiation and undifferentiation, advanced FIGO stage, severe muscular infiltration and positive lymphatic metastasis. miR-29a-3p was a tumor suppressor in EC. The up-regulation of miR-29a-3p distinctly reduced EC cells proliferation, colony formation, migration, invasion in vitro and growth in vivo. In previous researches, miR-29a-3p has been found to be acted as an important tumor suppressor in multiple human tumors [27, 28]. miR-29a-3p was lower expressed in laryngocarcinoma tissues than that in control tissues. It was a potential tumor-suppressive miRNA and suppressed the proliferation of laryngocarcinoma cells [11]. Additionally, miR-29a-3p inhibited the proliferation, migration and invasion of gastric cancer cells [14]. In cervical cancer, miR-29a-3p attenuated the proliferation and migration of tumor cells [29]. The inhibition of miR-29a-3p expression induced epithelial-mesenchymal transition in ovarian cancer cells [30]. Similarly, this study indicated that miR-29a-3p was a tumor suppressor in EC. It could be used as an effective candidate for EC target treatment.

VEGFA was identified as a direct target of miR-29a-3p in this study. VEGFA expression was negatively regulated by miR-29a-3p. VEGFA has been reported to be a target of miR-29a. miR-29a reduced the density of tumor microvessel in gastric cancer via targeting VEGFA [31]. In EC, miR-29b was found to inhibit angiogenesis by targeting VEGFA [32]. This study pointed out that VEGFA was directly bound to miR-29a-3p in the 3′-UTR region. VEGFA is a crucial invasion and metastasis factor for tumor metastasis. The reduction of VEGFA level is conductive to inhibit tumor cells invasion [31]. VEGFA up-regulation increased EC cells tumorigenicity in vivo, whereas VEGFA knockdown weakened EC cells proliferation, migration and invasion in vitro [33]. Hua et al. [34] illustrated the up-regulated VEGFA in EC tissues. VEGFA exacerbated the progression and metastasis of EC. This paper revealed that VEGFA up-regulation reversed the inhibitory effect of miR-29a-3p on EC cells in vitro malignant phenotype and growth in vivo.

**Fig. 8:**
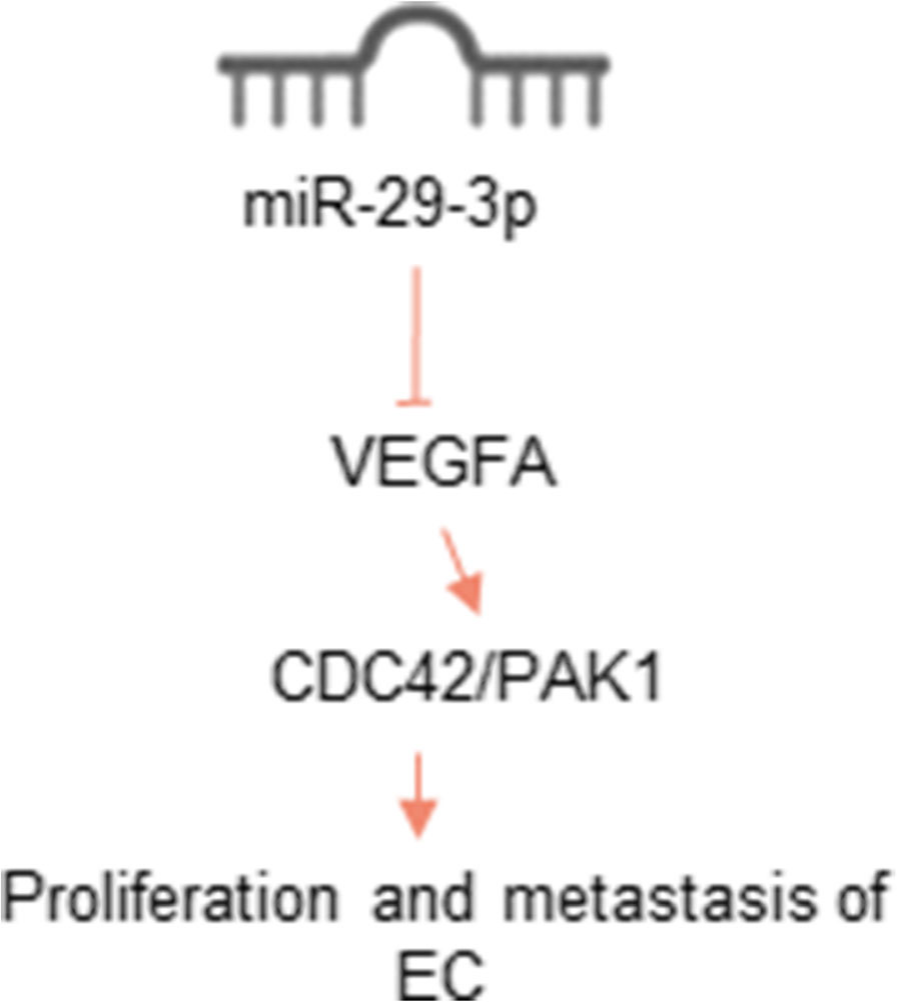
The flowchart of this research

This research monitored that miR-29a-3p inhibited the expression of CDC42 and the phosphorylation of PAK1. VEGFA up-regulation reversed the inhibition of miR-29a-3p on CDC42 expression and PAK1 phosphorylation. Recently, Chen et al. [35] have revealed that miR-29a suppressed cervical cancer cells proliferation and migration through inhibiting the CDC42/PAK1 signaling pathway. Dong et al. [36] researched that, miR-146a could inhibit cervical cancer cells colony formation, invasion and growth in vivo by inhibiting CDC42/PAK1 signaling pathway via targeting VEGF. Satterfield et al. [37] pointed out that CDC42/PAK1 was a cancer-promoting regulatory network in ewing sarcoma. miR-130b enhanced the malignant phenotype of ewing sarcoma cells by activating the CDC42/PAK1 signaling pathway. This article initially discovered that, miR-29a-3p might inhibit EC cells malignant development both in vitro and in vivo through inhibiting CDC42/PAK1 signaling pathway via directly targeting VEGFA. The flowchart was shown in Fig. 8. The discovery of this mechanism provided important theoretical basis for EC treatment with miR-29a-3p as the target.

## Conclusion

This study firstly studied the expression and function of miR-29a-3p in EC. The results suggested that miR-29a-3p was aberrantly down-regulated in EC. The abnormally reduced miR-29a-3p expression was associated with poor prognosis of EC patients. Up-regulation of miR-29a-3p could weaken EC cells proliferation, colony formation, migration, invasion in vitro and growth in vivo. Mechanically, miR-29a-3p might suppress the malignant development of EC by inhibiting CDC42/PAK1 signaling pathway via directly targeting VEGFA. These findings suggested that miR-29a-3p might be a promising target for EC treatment. This research provided important theoretical basis for EC treatment with miR-29a-3p as the target. Of course, more related research should be performed in the future in order to provide more accurate basis for the clinical use miR-29a-3p in EC target treatment.

## Supplementary Information

**Additional file 1.**

## Data Availability

All data generated or analysed during this study are included in this published article [and its supplementary information files].

## Abbreviations

EC: Endometrial cancer
miRNAs: MicroRNAs
VEGFA: Vascular endothelial growth factor A
HEC-1A and Ishikawa: Human EC cell lines
FBS: Fetal bovine serum
MTT: 3-(4,5-dimethylthiazol-2-yl)-2,5-diphenyltetrazolium bromide
DMSO: Dimethyl sulfoxide
OD: Optical density
PBS: Phosphate-buffered saline
Mut: Mutant type
Wt: Wild type
SDS-PAGE: Sodium dodecyl sulphate-polyacrylamide gel electrophoresis
PVDF: Polyvinylidene fluoride
ECL: Enhanced chemiluminescence
