# Supplementary material for: miR-29a-3p inhibits endometrial cancer cell proliferation, migration and invasion by targeting VEGFA/CD C42/PAK1
Source: BMC Cancer. 2021 Jul 21;21:843. doi: 10.1186/s12885-021-08506-z (PMC8293590; doi:10.1186/s12885-021-08506-z)

Fig 4 HEC-1A p-PAK1

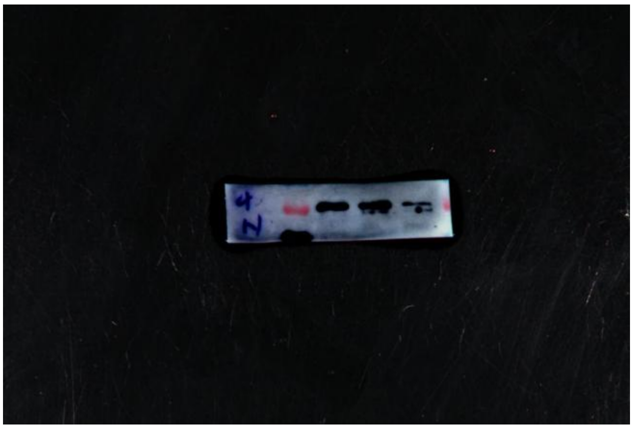

Fig 4 HEC-1A PAK1

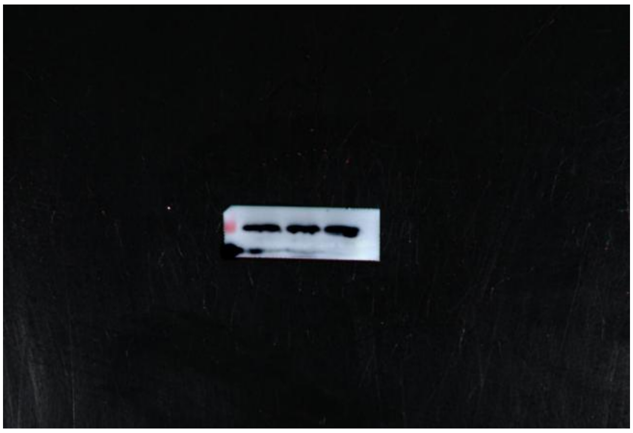

Fig 4 HEC-1A CDC42

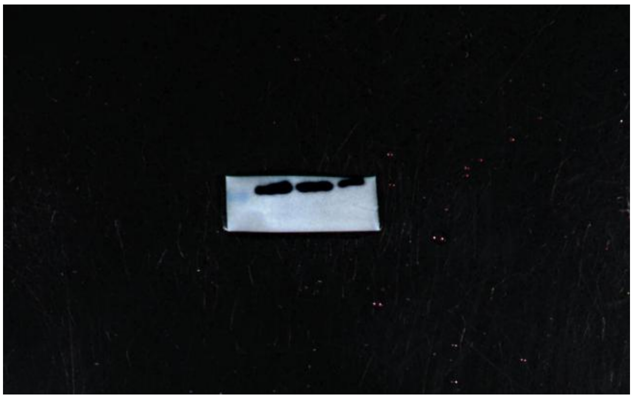

Fig 4 HEC-1A GAPDH

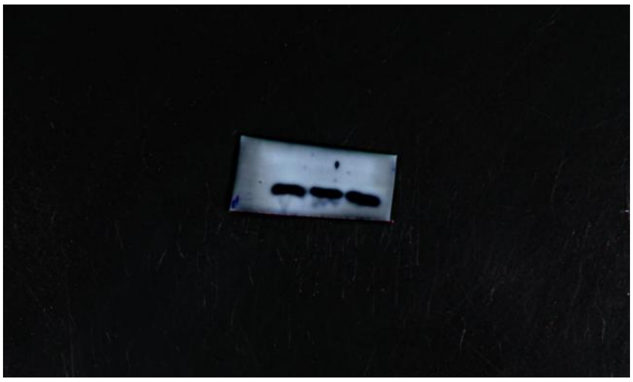

Fig 4 Ishikawa p-PAK1

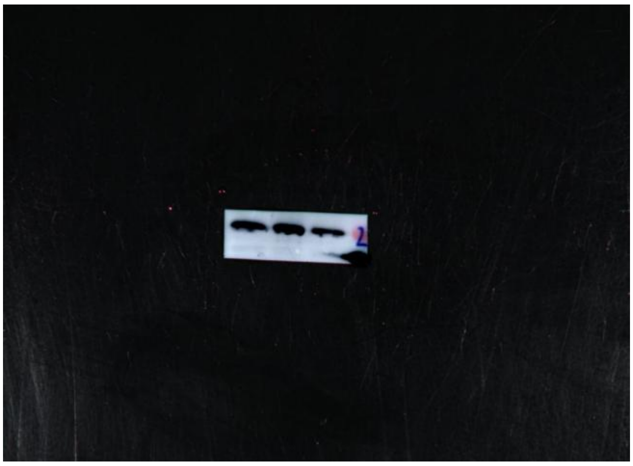

Fig 4 Ishikawa PAK1

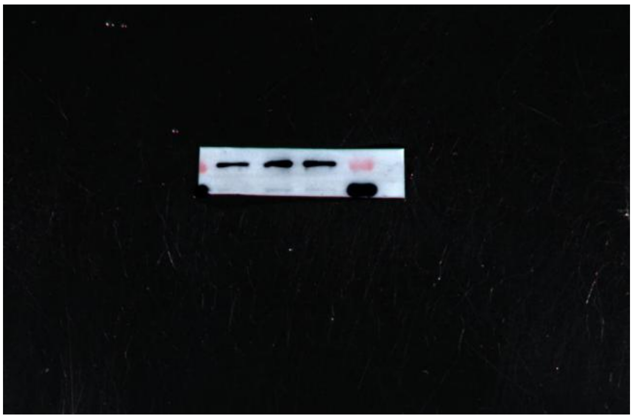

Fig 4 Ishikawa CDC42

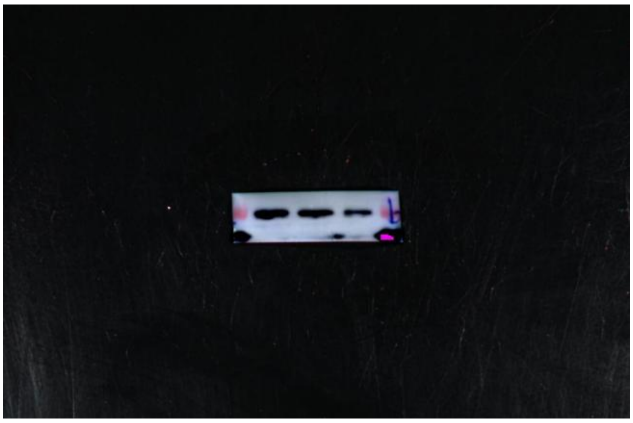

Fig 4 Ishikawa GAPDH

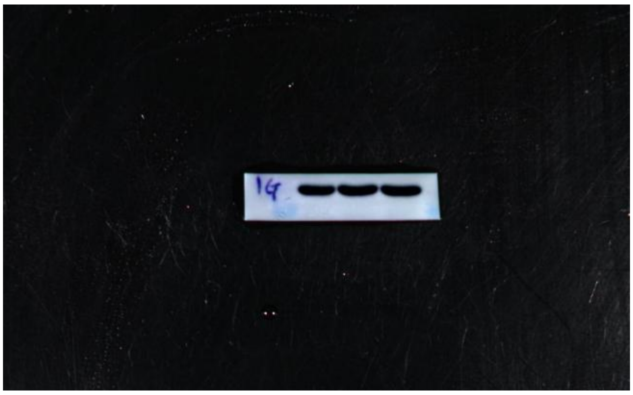

Fig 5 VEGF

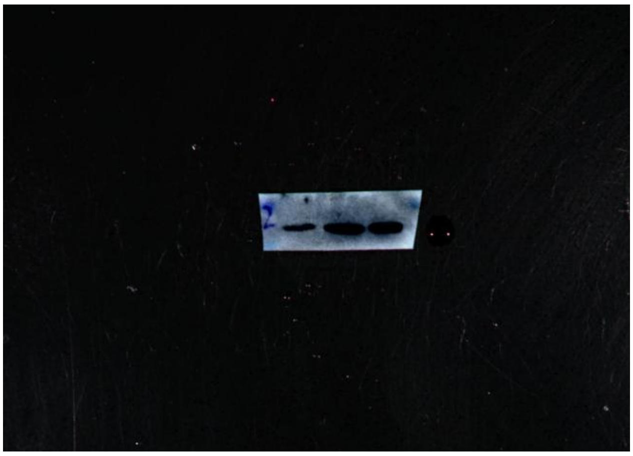

Fig 5 GAPDH

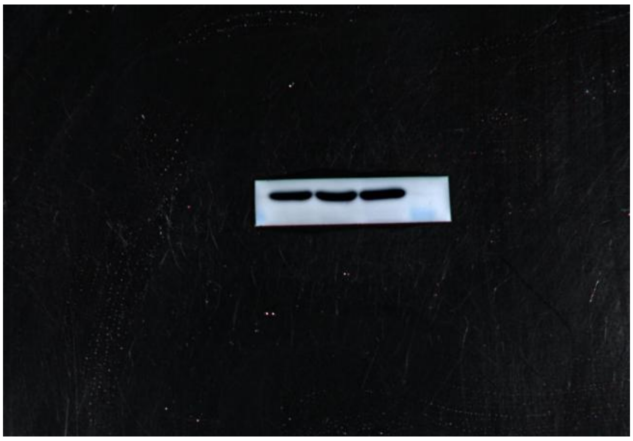

Fig 6 HEC-1A p-PAK1

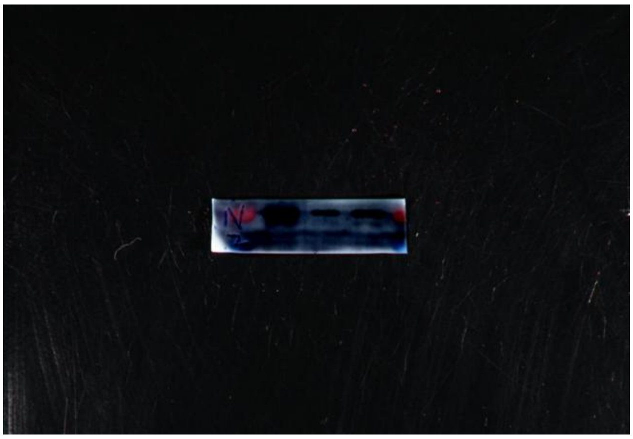

Fig 6 HEC-1A PAK1

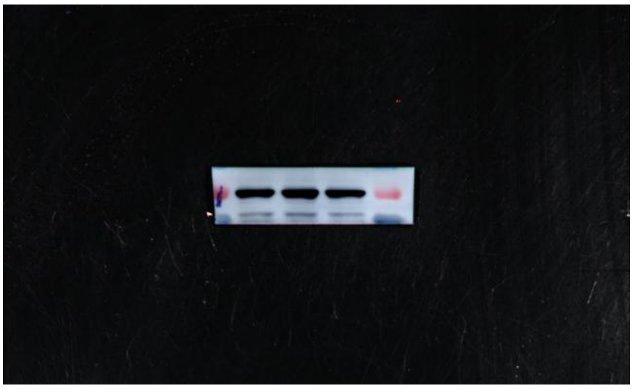

Fig 6 HEC-1A CDC42

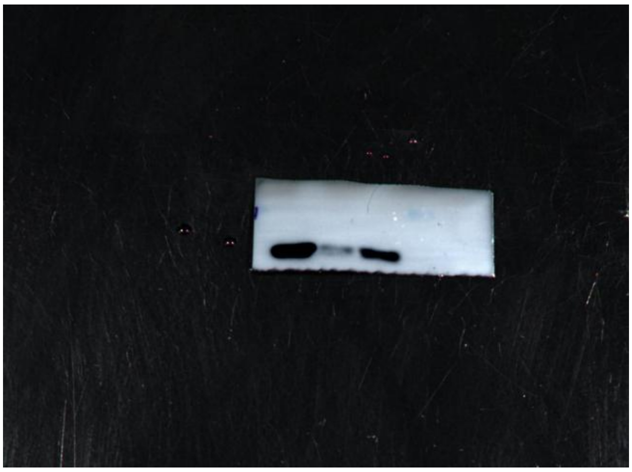

Fig 6 HEC-1A GAPDH

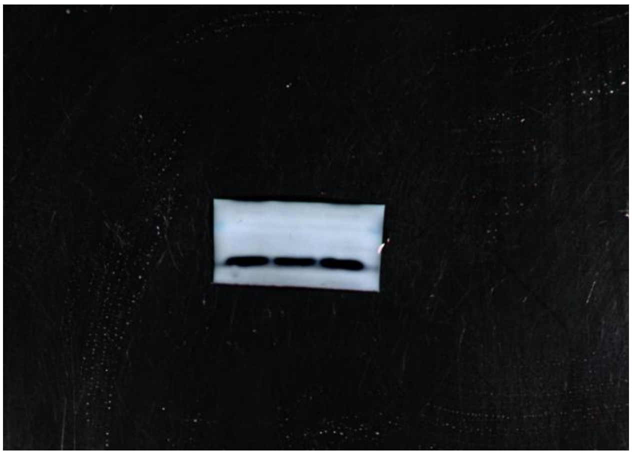

Fig 6 Ishikawa p-PAK1

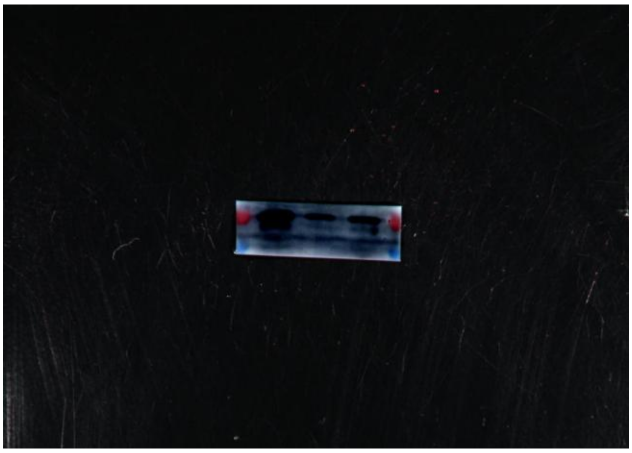

Fig 6 Ishikawa PAK1

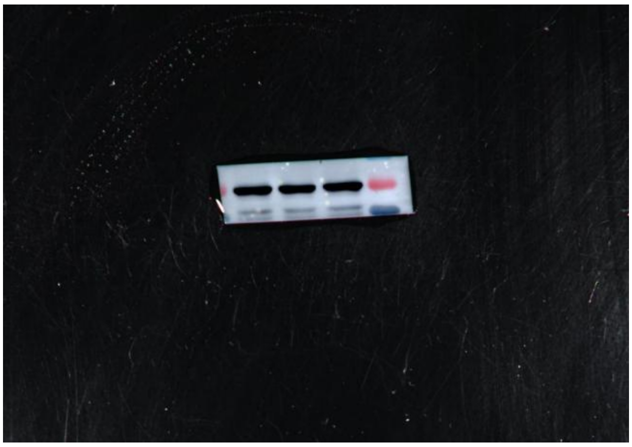

Fig 6 Ishikawa CDC42

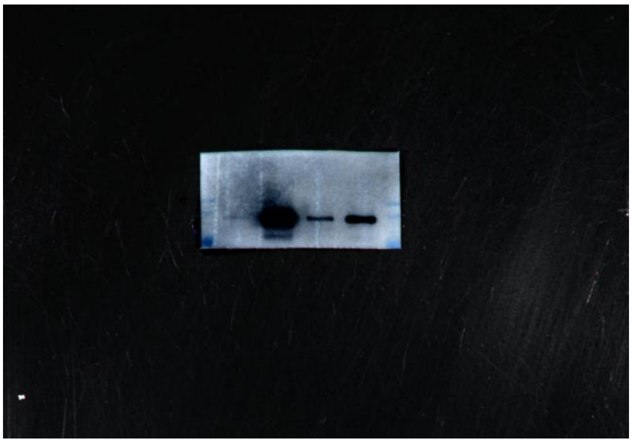

Fig 6 Ishikawa GAPDH

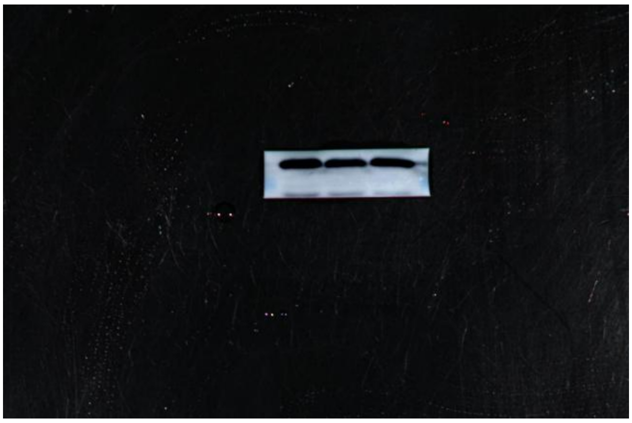

Fig 7 p-PAK1

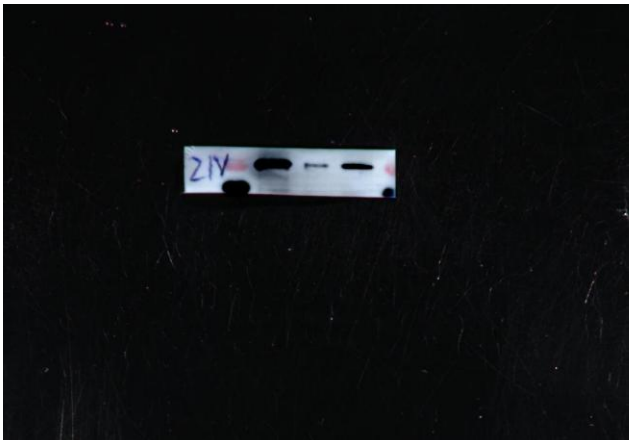

Fig 7 PAK1

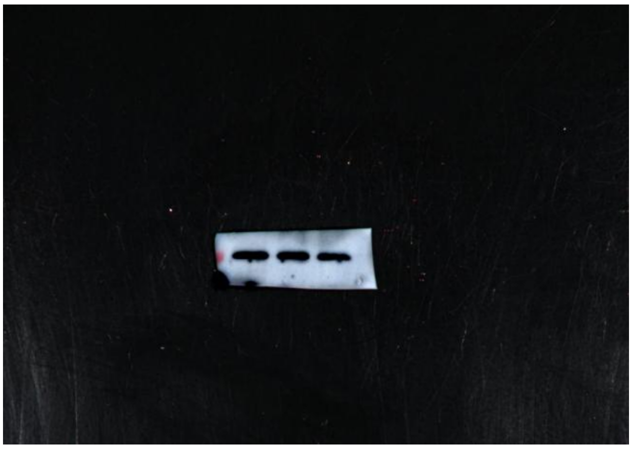

Fig 7 CDC42

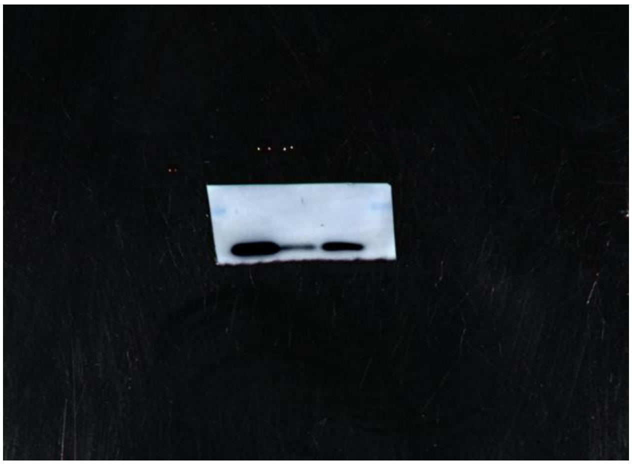

Fig 7 GAPDH

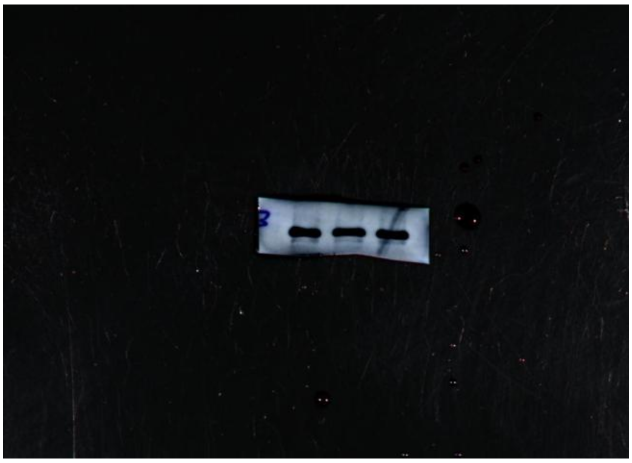

Supplement: Supplementary file 1 — Additional file 1. [file 12885_2021_8506_MOESM1_ESM.pdf]
